# Unusual Cause of Esophageal Obstruction in a Neonate Presenting as Esophageal Atresia

**Published:** 2013-10-01

**Authors:** Vijay C Pujar, Shirin S Joshi, Sangappa M Dhaded

**Affiliations:** Department of Pediatric Surgery, KLE University, Belgaum, Karnataka, India; 1 Department of Pediatrics KLE University, Belgaum, Karnataka, India

**Keywords:** Neonate, Foreign Body, Esophagus

## Abstract

Esophageal atresia is the commonest cause of obstruction to esophageal lumen in neonates. Foreign bodies in newborns are extremely rare. We report a rare case of esophageal obstruction closely mimicking atresia due to foreign bodies inserted in a female neonate with homicidal intension.

## INTRODUCTION

Foreign body (FB) ingestion is a common and serious problem in pediatric population between 6 months to 3 years of age. Coins are the most common foreign body ingested by children. Various other FB like stone, ornament, ring, button and safety pin have also been reported. Esophageal FB are extremely rare in neonates and can be life-threatening. We report a neonate where unusual non-metallic FB led to a presentation akin to that of an esophageal atresia. 

## CASE REPORT

A 6-day-old full term female baby, delivered at a peripheral hospital, was brought with continuous frothing of saliva from the mouth, refusal of feeds and a progressive respiratory distress since birth. No records were available regarding the initial resuscitation of the baby and whether a nasogastric tube was passed at birth to assess esophageal patency. She was seen at three different hospitals where a diagnosis of esophageal atresia was made before she reached our center for further management.

On examination, the baby was a sick-looking full-term female neonate having severe tachypnea with persistent white froth in mouth. No chest wall retractions or central cyanosis was noted. Heart rate was 162 beats per minute and respiratory rate was 54 per minute. Auscultation of the chest revealed equal air entry on both sides with coarse crepitations. Insertion of a red rubber tube through the mouth revealed obstruction at 10cms from the mouth was noted. Chest roentgenogram with the red rubber tube in situ demonstrated the tube lying at the level of the third thoracic vertebrae with presence of air in the stomach. Echocardiographic examination was normal. A clinical diagnosis of esophageal atresia with distal tracheo-esophageal fistula (Vogt type III) was made. She was stabilized by low-pressure continuous upper pouch suction, oxygen, antibiotics and chest physiotherapy.

The patient was taken up for a right postero-lateral thoracotomy. On exploration, the esophagus was found to be in continuity with no evidence of esophageal atresia. Careful palpation of the esophagus revealed a hard mass in the upper esophagus. Longitudinal esophagotomy over the mass showed multiple foreign bodies (beetle nuts pieces) and they were meticulously removed (Fig. 1). After removal of beetle nut pieces, a nasogastric tube could easily be passed into the stomach from the mouth. Esophagus was repaired over the nasogastric tube and chest was closed after putting an intercostal drain. Post-operative period was uneventful.

**Figure:**
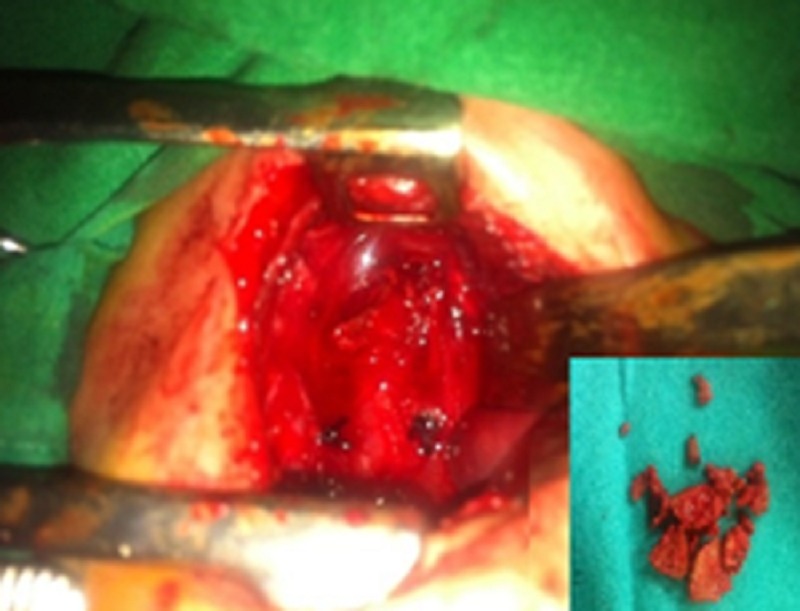
Figure 1: Intra-operative photograph revealing intact esophagus; inset shows extracted beetle nut pieces.

On retrospective enquiry to understand the motive, we found that the baby was born on a supposedly inauspicious birth star and was believed to be a harbinger of bad luck due to a misguided belief in an astrologer. The grandfather of the baby had forcefully pushed small pieces of beetle nut down the child’s throat. 

## DISCUSSION

Occurrence of FB ingestion in neonates is rare with only a few reported cases in literature. [1, 2] Small ornaments and button batteries have been described in neonates, which have been inserted playfully by an elder sibling or due to a homicidal attempt on an unwelcome female baby. [3, 4] Aggarwal and Gupta reported a case of retained cotton swab in the esophagus probably retained during finger sweep during resuscitation. [5]

Respiratory distress is the most common manifestation of an FB in esophagus in neonates. Respiratory distress along with failure to pass a stiff catheter poses a problem in ruling out esophageal atresia, as in our case. [5, 6] Availability of proper history and visualization on radiograph of the chest should help in clinching the diagnosis. However, nonavailability of proper history and a radiolucent FB poses problems in diagnosis and management, as in our case. Non-visualization of fistula on preoperative bronchoscopy may also point towards an alternate diagnosis especially when radiographs demonstrate the presence of ample gas in the stomach. Computerized tomography has also been used in diagnosing esophageal foreign bodies in neonates. [5]

Primary Endoscopic removal is recommended if the pre-op diagnosis is available. [5,7] Intraoperative removal by rigid esophagoscopy can also be utilized whenever available and would avoid the need for an esophagotomy. [5] Cervical esophagotomy and thoracotomy have been performed in undiagnosed or impacted cases. [4]

To conclude, neonatal esophageal FB may mimic esophageal atresia and a high index of suspicion is required for a proper diagnosis and treatment. 

## Footnotes

**Source of Support:** Nil

**Conflict of Interest:** None

